# Interpretation of gut microbiota data in the ‘eye of the beholder’: A commentary and re‐evaluation of data from ‘Impacts of radiation exposure on the bacterial and fungal microbiome of small mammals in the Chernobyl Exclusion Zone’

**DOI:** 10.1111/1365-2656.13667

**Published:** 2022-06-13

**Authors:** Phillip C. Watts, Tapio Mappes, Eugene Tukalenko, Timothy A. Mousseau, Zbyszek Boratyński, Anders P. Møller, Anton Lavrinienko

**Affiliations:** ^1^ Department of Biological and Environmental Science University of Jyväskylä Jyväskylä Finland; ^2^ National Research Center for Radiation Medicine of the National Academy of Medical Science Kyiv Ukraine; ^3^ Department of Biological Sciences University of South Carolina Columbia South Carolina USA; ^4^ CIBIO/InBio, Research Centre in Biodiversity and Genetic Resources University of Porto Vairão Portugal; ^5^ Laboratoire d’Ecologie, Systématique et Evolution CNRS UMR 8079, Université Paris‐Sud 11 Orsay Cedex France

**Keywords:** amplicon sequencing, diet, microbiota, mycobiota, radiation effects

## Abstract

1. Evidence that exposure to environmental pollutants can alter the gut microbiota composition of wildlife includes studies of rodents exposed to radionuclides.
2. Antwis et al. (2021) used amplicon sequencing to characterise the gut microbiota of four species of rodent (*Myodes glareolus*, *Apodemus agrarius*, *A. flavicollis* and *A. sylvaticus*) inhabiting the Chernobyl Exclusion Zone (CEZ) to examine possible changes in gut bacteria (microbiota) and gut fungi (mycobiota) associated with exposure to radionuclides and whether the sample type (from caecum or faeces) affected the analysis.
3. The conclusions derived from the analyses of gut mycobiota are based on data that represent a mixture of ingested fungi (e.g. edible macrofungi, polypores, lichens and ectomycorrhizae) and gut mycobiota (e.g. microfungi and yeasts), which mask the patterns of inter‐ and intraspecific variation in the authentic gut mycobiota.
4. Implying that ‘faecal samples are not an accurate indicator of gut composition’ creates an unnecessary controversy about faecal sampling because the comparison of samples from the caecum and faeces confounds many other possible drivers (including different animals from different locations, sampled in different years) of variation in gut microbiota.
5. It is relevant also that Antwis et al.'s (2021) data lack statistical power to detect an effect of exposure to radionuclides on the gut microbiota because (1) all of their samples of *Apodemus* mice had experienced a medium or high total absorbed dose rate and (2) they did not collect samples of bank voles (*M. glareolus*) from replicate contaminated and uncontaminated locations.
6. Discussion of Antwis et al.'s (2021) analysis, especially the claims presented in the Abstract, is important to prevent controversy about the outcome of research on the biological impacts of wildlife inhabiting the CEZ.

## Graphical abstract

The authors' work highlights: (1) the need to consider host and fungal ecology when analysing the apparent assemblages of fungi from animal gastrointestinal tracts, and (2) that differences in sampling and laboratory protocols need to be fully considered when making a contrast between separate studies of wildlife gut microbiota. Photo credit: Pinja Rautio.

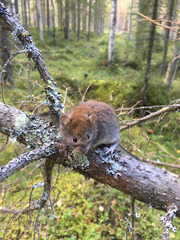

## 1 INTRODUCTION

Animal gut microbial communities provide essential services for their host, such as interacting with the host’s immune system (Pickard et al., 2017; Round & Mazmanian, 2009), defending against pathogen invasion (Rosshart et al., 2019; Pickard et al., 2017) and processing dietary material to provide important metabolites (Morrison & Preston, 2016; Sonnenburg & Bäckhed, 2016). As disruption to the gut microbiota can affect the health of the host, there is much interest in identifying features of the host or its environment that can impact the gut microbiota community composition (Zaneveld et al., 2017). Infection by parasites or pathogens (Kreisinger et al., 2015; Sabey et al., 2021), the level of biodiversity or habitat disturbance (Barelli et al., 2020), changes in diet and season (Guo et al., 2021; Lavrinienko et al., 2020; Maurice et al., 2015) and exposure to pollutants (Brila et al., 2021) are associated with a change in the gut microbiota of wildlife.

Evidence that exposure to pollutants impacts the gut microbiota extends to rodents inhabiting areas contaminated by radionuclides (Lavrinienko et al., 2020; Lavrinienko, Mappes, et al., 2018; Lavrinienko, Tukalenko, et al., 2018). Antwis et al. (2021) characterised the gut microbiota of four species of rodent inhabiting the Chernobyl Exclusion Zone (CEZ), a region surrounding the former nuclear power plant at Chernobyl, Ukraine, where substantial areas are contaminated by radionuclides. Samples were collected from bank voles (*Myodes glareolus*), where animals were caught from locations that represented a gradient of contamination, and from three species of mice (*Apodemus agrarius*, *A. flavicollis* and *A. sylvaticus*), where animals had experienced ‘medium’ (4–42 μGy/hr) or ‘high’ (>42 μGy/hr) absorbed dose rates. The CEZ was established to limit human exposure to radionuclides, but the wildlife inhabiting the CEZ provide the best‐studied models of the biological impacts of exposure to environmental radionuclides (Beresford et al., 2016; Møller & Mousseau, 2006; Mousseau, 2021). Despite decades of research on wildlife within the CEZ, the effects of exposure to environmental radionuclides in wildlife remain a source of controversy (Beresford et al., 2016; Beresford, Horemans, et al., 2020; Kesäniemi et al., 2018; Møller & Mousseau, 2006; Mousseau, 2021).

In their Abstract, Antwis et al. (2021) conclude

(1a) ‘We provide novel evidence that host species determines fungal community composition’,
(1b) ‘… fungi (Steccherinaceae and Strophariaceae) in the guts of bank voles that may serve as biomarkers of radiation exposure’,
(2) ‘…considerable variation between results for faecal and gut samples of bank voles, suggesting faecal samples are not an accurate indicator of gut composition’ and
(3) ‘associations between radiation exposure and microbiome composition of gut samples were not robust against geographical variation’.

These points have clear implications for our understanding of the effects of environmental radionuclides on wild animal gut microbiota, and for studies of exposure to pollutants on wildlife microbiota generally. However, statements (1a, b) need clarification as the fungal amplicon sequence data represent a mixture of ingested fungal material and authentic gut mycobiota. Conclusion (2) creates an unnecessary controversy as it neglects to account for confounding effects of multiple drivers of variation in the gut microbiota in the comparison of samples from the caecum and faeces. We discuss statement (3) to highlight the need for greater clarity about the power of Antwis et al.'s (2021) study design to identify the effects of exposure to radionuclides on the gut microbiota of small rodents.

## 2 MATERIALS AND METHODS

**FIGURE 1:**
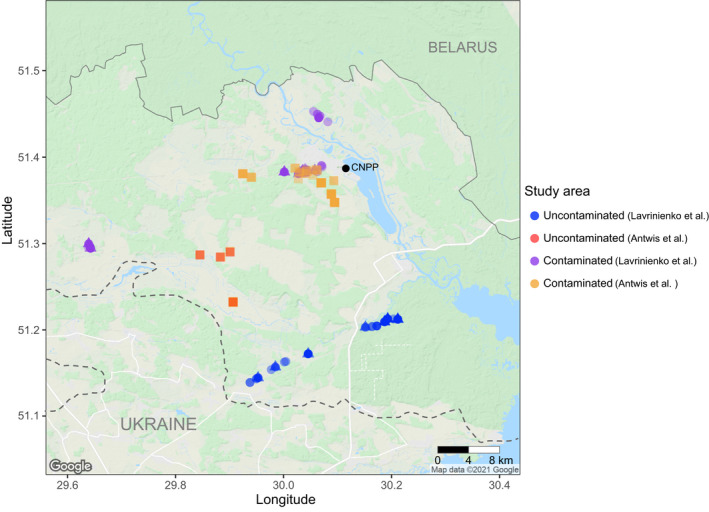
Distribution of samples of rodent gut microbiota from within the Chernobyl Exclusion Zone (CEZ) collected by Antwis et al. (2021) and Lavrinienko, Mappes, et al. (2018), Lavrinienko et al. (2020) (note that Lavrinienko, Mappes, et al. 2018 also collected microbiota samples from two locations outside the CEZ [~80 km south, near Kyiv]) and these data are not shown in this figure. Dashed line represents the border around the CEZ in Ukraine (area ~2,050 km^2^). Shapes indicate trapping locations used in Lavrinienko, Mappes, et al. (2018, circles), in Lavrinienko et al. (2020, triangles) and in Antwis et al. (2021, squares). The figure was created using the ggmap (https://github.com/dkahle/ggmap) package in R

Antwis et al. (2021) used amplicon sequencing to characterise the bacterial and fungal components of the gut microbiota in four species of rodent (bank vole *M. glareolus*, wood mouse *Apodemus sylvaticus*, striped field mouse *A. agrarius* and yellow‐necked mouse *A. flavicollis*) inhabiting the CEZ, Ukraine. Samples were collected by live trapping from two areas: (1) a contaminated site in the Red Forest and adjacent area, and (2) an uncontaminated site about 10 km south‐west of the Red Forest (Figure 1). We re‐analysed some of the amplicon sequence data used by Antwis et al. (2021) to examine the potential proportion of non‐resident fungal sequence variants (SVs) in their data. As the data used in this article were downloaded from a public archive, we did not seek permissions for fieldwork or ethical approval for the work.

Full details about the sample data are provided in the original publication (Antwis et al., 2021). Briefly, data were obtained from GenBank (PRJNA594002) and processed in QIIME2 v.2020.6 (Bolyen et al., 2019), using CUTADAPT (https://github.com/marcelm/cutadapt) to remove primer/adaptor sequences and DADA2 (Callahan et al., 2016) to denoise the data. Taxonomy for SVs was assigned using the SKLEARN machine learning taxonomy classifier (Bokulich et al., 2018) against the UNITE v.8 (Nilsson, Larsson, et al., 2019) reference database. Because there are no reliable data that identify all species of fungi that could be ingested (either by direct consumption, or by association with other components of the diet) by bank voles and *Apodemus* mice in the CEZ, we used informed filtering to identify likely non‐resident fungal SVs (see Lavrinienko, Scholier, et al., 2021). SVs were assigned to the major classes of microfungi using the information at the Microfungi Collections Consortium (www.microfungi.org/table1). We further classified fungal SVs according to guild or growth form based on assignments made by FUNGUILD v.1.2 (Nguyen et al., 2015). SVs categorised as plant pathogens, epi‐ and endophytes, lichens, mycorrhizae and wood saprophytes were assumed to be part of the ingested, non‐resident fungal material in the gut, as were SVs with large fruiting bodies/growth forms (e.g. as gasteroid, pezizoid, tremelloid, etc.), which left the remaining microfungi and yeasts (and taxa with unclear growth forms but which were assigned as animal pathogens or SVs that lacked information about guild, e.g. because taxonomic resolution was not assigned below Phylum level) as candidate resident gut mycobiota. Thus, we make a contrast between data that can represent dietary items (principally macrofungi and lichens, plant‐associated fungal pathogens, mycorrhizae or endophytes) and the remaining data as a candidate resident gut mycobiota (many microfungi and yeasts, taxa associated with animals and poorly known fungi).

Data were imported into PHYLOSEQ (McMurdie & Holmes, 2013) for analyses in R v.4.0.5 (R Core Team, 2020). Fungal data were rarefied to an even depth of 5,000 (all SV data, that resulted in a loss of 11 samples and 1,020 SVs), 3,000 (possible resident gut fungi, with a loss of 27 samples and 980 SVs) or 1,000 (possible dietary and diet‐associated fungi, with a loss of 21 samples and 710 SVs) reads per sample. We calculated alpha diversity (observed number of SVs) and beta diversity (Bray–Curtis dissimilarity) in phyloseq. Variation in alpha diversity was assessed using pairwise Wilcoxon rank‐sum tests with Holm correction. The ADONIS2 function in vegan (Oksanen et al., 2020) was used to examine the amount of variation in beta diversity explained by features of the data, such as host species (bank vole, wood mouse, striped field mouse and yellow‐necked mouse), sampling year (2017, 2018) and total absorbed dose rate of radiation (μGy/hr). BETADISP function in vegan (Oksanen et al., 2020) was used to determine whether there were significant differences in dispersion among groups of samples.

## 3 RESULTS

### 3.1 Composition of the community of fungi detected in rodent guts

**FIGURE 2:**
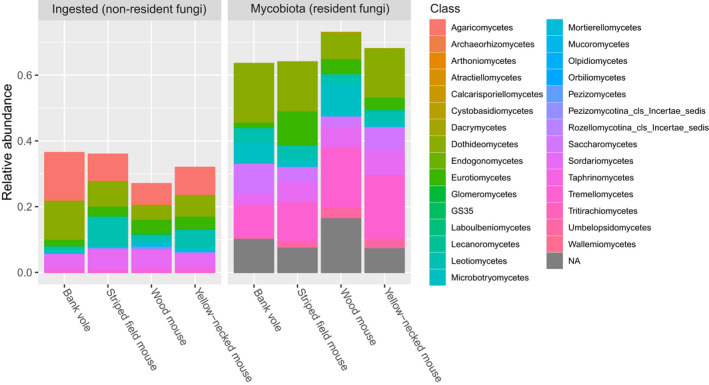
Proportions of fungal classes identified in the gut and faecal samples from four species of rodent, separated by their possible resident (mycobiota) or non‐resident (ingested) status in the host’s gastrointestinal tract

By classifying probable ingested fungi as macrofungi, plant pathogens, endophytic species and taxa associated with decaying wood, we separated the fungal SVs in rodent gut samples into 2,608 putative gut residents and 1,566 possible non‐residents. This filtering procedure indicates that about a third of the read data could be derived from ingested fungi (Figure 2), with about 10%–15% of the reads assigned to macrofungi (e.g. Agaricomycetes).

**FIGURE 3:**
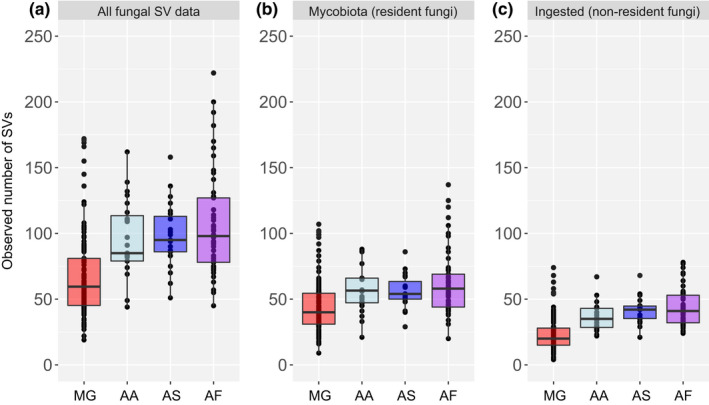
Effect of filtering fungal sequence variants (SVs) by their traits on alpha diversity (observed number of SVs) of the assemblage of gut fungi present in caecum and faeces in four species of rodent. (a) All SV data, (b) the likely resident gut fungi (mycobiota) and (c) the possible fungal SVs that were ingested as part of the host’s diet (non‐resident fungi). Red, bank vole (*Myodes glareolus*, MG); light blue, striped field mouse (*Apodemus agrarius*, AA); blue, wood mouse (*A. sylvaticus*, AS); purple, yellow‐necked mouse (*A. flavicollis*, AF)

**FIGURE 4:**
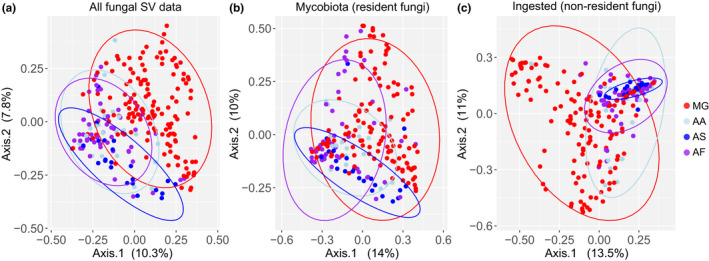
Effect of filtering fungal sequence variants (SVs) by their traits on the apparent pattern of interspecific differences in fungal assemblage present in rodent caecum and faecal samples. (a) All SV data, (b) the likely resident gut fungi (mycobiota) and (c) the possible fungal SVs that were ingested as part of the host’s diet (non‐resident fungi). Red, bank vole (*Myodes glareolus*, MG); light blue, striped field mouse (*Apodemus agrarius*, AA); blue, wood mouse (*A. sylvaticus*, AS); purple, yellow‐necked mouse (*A. flavicollis*, AF). Ordination is a principal coordinate analysis (PCoA) based on Bray–Curtis dissimilarities

The inclusion of all fungal SV data affects the analyses. Although interspecific differences in alpha diversity are apparent in unfiltered and filtered datasets, with bank voles having significantly fewer fungal SVs than species of *Apodemus* (pairwise Wilcoxon rank‐sum test with Holm correction, *p* < 0.05 for all comparisons; Table 1, Figure 3), the inclusion of dietary material inflates the estimates of alpha diversity (Figure 3). Filtering the fungal SVs also impacts the pattern of beta diversity. Without SV filtering, bank voles differ from the three species of *Apodemus* mice, with samples from wood mice being somewhat different to those from striped field mice and yellow‐necked mice (Figure 4a). There appears to be less interspecific variation in the gut mycobiota (Figure 4b) than among the ingested fraction of fungi, which is characterised by high inter‐individual variation among samples of bank voles and little inter‐individual variation among the samples of wood mice (Figure 4c). Filtering the SVs affects the explanatory power of ‘host species’ with slightly less interspecific variation among the samples of possible resident gut fungi (cf. values of *R* ^2^ in Table 2). Moreover, significant (*p* = 0.001) interspecific differences in the amount of dispersion were detected in the analyses based on the entire dataset and the suspected non‐resident fungi, but not in the subset of possible resident gut fungi (Table 2, Figure 4).

**TABLE 1:** Wilcoxon rank test probabilities (with Holm correction) for differences in alpha diversity (observed number of SVs) of fungal SVs identified in the caecum and faeces of four species of rodent. (a) All data, (b) the likely resident gut fungi (mycobiota) and (c) the possible fungal SVs that were ingested as part of the host’s diet (non‐resident fungi)

	Bank vole	Striped field mouse	Wood mouse
(a) All fungi			
Striped field mouse	2.8e‐05		
Wood mouse	3.9e‐06	0.60	
Yellow‐necked mouse	3.1e‐11	0.60	0.68
(b) Mycobiota			
Striped field mouse	0.0039		
Wood mouse	0.0018	1.00	
Yellow‐necked mouse	3.2e‐06	1.00	1.00
(c) Ingested fungi			
Striped field mouse	2.4e‐06		
Wood mouse	2.3e‐08	0.12	
Yellow‐necked mouse	1.1e‐15	0.10	0.94

**TABLE 2:** Effect of filtering fungal sequence variants (SVs) by their traits on the level of variation in beta diversity and dispersion in amplicon sequence data from samples of caecum and faeces from four species of rodent. (a) All data, (b) the likely resident gut fungi (mycobiota) and (c) the possible fungal SVs that were ingested as part of the host’s diet (non‐resident fungi)

Beta diversity						Dispersion					
	*df*	SS	*R* ^2^	*F*	*p*		*df*	SS	*R* ^2^	*F*	*p*
(a) All fungi											
Species	3	6.634	0.072	6.160	0.001	Species	3	0.092	0.031	6.644	0.001
Residual	237	85.078	0.928			Residual	237	1.089	0.005		
Total	240	91.712	1.000								
(b) Mycobiota											
Species	3	5.695	0.070	5.512	0.001	Species	3	0.058	0.019	2.169	0.102
Residual	221	76.117	0.930			Residual	221	1.961	0.009		
Total	224	81.812	1.000								
(c) Ingested fungi											
Species	3	7.745	0.088	7.264	0.001	Species	3	0.187	0.062	12.051	0.001
Residual	227	80.676	0.912			Residual	227	1.176	0.005		
Total	230	88.421	1.000								

## 4 DISCUSSION

### 4.1 Composition of the community of fungi detected in rodent guts

With our understanding of the processes that affect wild animal gut fungi (gut mycobiota) limited (Huseyin et al., 2017; Kong & Morris, 2017; Nilsson, Anslan, et al., 2019), it is important to carefully consider the possible sources of fungal material in samples from the animal gut (Lavrinienko, Scholier, et al., 2021). As amplicon sequencing enumerates all types of DNA (for the target region and taxon, such as a partial region of the 16S rRNA for bacteria or the ITS for fungi; Knight et al., 2018; Lavrinienko, Jernfors, et al., 2021) within a sample, the resulting SVs are derived from the authentic gut residents and any non‐resident (ingested) material. A combination of (1) comparatively few fungal cells (compared with bacterial cells) in the vertebrate gut (Qin et al., 2010, Iliev et al., 2012) and (2) ingestion of fungi by many animals, for example by consumption of macrofungi or lichens (Abt & Bock, 1998; Fogel & Trappe, 1978) or intake of fungal plant pathogens, commensals/symbionts or the microfungi in fermenting or decaying material, raises the potential that amplicon sequencing‐based studies of gut mycobiota will contain a substantial amount of non‐resident gut fungi (Lavrinienko, Scholier, et al., 2021).

Antwis et al. (2021) did not differentiate among resident gut mycobiota or ingested fungal material. Evidence for an abundant fraction of non‐resident fungal material in Antwis et al.'s (2021) analysis of ‘gut mycobiota’ is derived from their potential ‘biomarkers of radiation exposure’: the Steccherinaceae (members of the Polyporales that are a cause of white rot, often growing on wood) and the Strophariaceae (saprophytes within the Agaricales). As macrofungi associated with decaying material, these taxa are unlikely to be long‐term residents of the mammalian gut. Our re‐analysis of Antwis et al.'s (2021) data identified a substantial fraction of likely dietary fungi in their samples, including members of the Agaricomycetes that are often actively selected for ingestion by rodents (Abt & Bock, 1998; Fogel & Trappe, 1978). By re‐analysing Antwis et al.'s (2021) data, we show how (a lack of) filtering the fungal SVs can impact the outcomes of analyses of gut mycobiota (via alpha diversity, beta diversity and dispersion) and hence the study conclusions. For example, there is a clear possibility that radiation exposure impacts the diversity of fungi or other food items in the environment that, in turn, affects the host’s (dietary) intake of non‐resident gut fungi, in addition, or alternate, to, any direct effect of the environment on the rodent gut mycobiota per se. Amplicon sequencing‐based analyses of wildlife gut mycobiota thus require an assessment of the ecology of host(s) and fungi to identify the possible source(s) of fungal SVs and make better informed conclusions about ecological and evolutionary drivers of inter‐ and intraspecific differences in gut mycobiota (Lavrinienko, Scholier, et al., 2021).

### 4.2 Differences between microbiota detected in samples from faeces and the caecum

The extent to which sample type or laboratory procedures impact amplicon sequence data is an important issue for microbiota research (Knight et al., 2018; Panek et al., 2018). By describing a caecum sample as providing ‘…data on the true gut microbiome’, Antwis et al. (2021) create an artificial impression about data quality in microbiota studies as one may define, for example, a community of microbes from the stomach, small intestine, caecum, colon and/or faeces (Kreisinger et al., 2015; Martinez‐Guryn et al., 2019; Videvall et al., 2018), and make a distinction between the lumen and the gut mucosa (Albenberg et al., 2014). Alternative sampling locations of the gut microbiota are neither ‘true’ nor ‘false’ but are different representations of natural variation in microbial assemblages that accompany the process of digestion and changes in the environment (e.g. pH, oxygen tension, flow rate, etc., Martinez‐Guryn et al., 2019) along the gastrointestinal tract. While the composition of microbes in faecal samples often (Ingala et al., 2018; Velasco‐Galilea et al., 2018; Videvall et al., 2018; Zhou et al., 2020) differs from the microbes identified from regions of the digestive tract, the comparison itself depends on the regions of the gastrointestinal tract under consideration; for instance, faeces may recapitulate the microbiota of the colon, but not ileum or caecum (Videvall et al., 2018). Moreover, when conducted with great care (e.g. standardising the time between defecation and sample collection, and using consistent and appropriate conditions to store the samples), faecal sampling is an invaluable, non‐destructive method of obtaining a representative sample of the gut microbiota that allows (and is a unique option for) longitudinal sampling (Antwis et al., 2019; Johnson et al., 2019; Lavrinienko et al., 2020).

The relevant issue about sample type is whether it interacts with the treatment to produce a biased outcome. Antwis et al. (2021) state ‘For bank voles, we observed differences in microbial communities associated with the gut and faeces, …. We also observed significant differences in the relationships between radiation and gut/faecal microbial families’. Unfortunately, Antwis et al. (2021) did not use an appropriate study design that compares paired samples of faeces and caecum from the same individuals collected at a similar time (cf. Ingala et al., 2018; Velasco‐Galilea et al., 2018; Videvall et al., 2018; Zhou et al., 2020). Rather, the variation in microbiota that Antwis et al. attribute to ‘sample type’ (faeces or caecum) is confounded with variation derived from samples being collected from (1) different animals, (2) different years, (3) different time points (July/August versus August) and (4) different locations and hence from samples (5) that differ in absorbed dose rates. As habitat, host genetics and season, etc. associate with variation in gut microbiota (Bonder et al., 2016; Lavrinienko et al., 2020; Li et al., 2019; Maurice et al., 2015; Park et al., 2019), the roughly 1% of variation in beta diversity attributed to ‘sample type’ could support the conclusion that sample type itself has little impact on microbiota composition in these data. Antwis et al. (2021) acknowledge that ‘bank vole gut samples were collected in 2018 from across the CEZ, whereas the faeces samples collected in 2017 were all from inside the Red Forest (including from a number of sites that had been recently burnt), which may also be influencing the observed differences between the gut and faecal samples’. However, the authors did not consider the other relevant issues that (1) only 22 samples of bank vole faeces were collected in contrast to the 132 samples from the bank vole caecum and (2) that all of the bank vole faecal samples were taken from animals that had received a high absorbed dose rate (>70 μGy/hr) in contrast to the caecum samples that were sourced from animals that had received low (<4 μGy/hr), medium (4–42 μGy/hr) and high (>42 μGy/hr) total absorbed dose rates. With a marked difference in sample size and in the range of absorbed dose rates among faecal and caecum samples, it is unsurprising that the different sample types yielded different statistical relationships between absorbed dose rate and gut microbiota.

### 4.3 Associations between radiation exposure and gut microbiota composition

Antwis et al. (2021) note that associations between gut microbiota composition were not robust when the analyses were controlled for geographic distance, with sampling site explaining some variation in bacterial beta diversity. Hence, they conclude that ‘any variation in microbiome composition arising from proximity to the Chernobyl Nuclear Power Plant is more likely a habitat effect than a result of radiation exposure’. Because bank voles can disperse several kilometres per year (White et al., 2012), Antwis et al.'s (2021) animals with high absorbed dose rates represent samples from one cohort of animals within a single contaminated area (the Red Forest locality) rather than from separate cohorts inhabiting the different contaminated sites within the CEZ (see Figure 1). A lack of replication of sites with similar dose rate categories is a curious design for a study of radiation effects on wildlife, especially when the focal contaminated area is the Red Forest as this location is argued to comprise poor habitat (Beresford, Scott, et al., 2020). Studies of radiation effects that do not employ a replicated study‐site design confound the treatment (radiation exposure) with location and thus are somewhat destined to support the idea that exposure to radionuclides has no detrimental biological impacts: a lack of statistical effect can be interpreted that radiation exposure has little biological impact, while any apparent biological impacts can be dismissed as location‐specific effects (e.g. the poor‐quality habitat in the Red Forest) rather than exposure to radionuclides! Only by collecting samples from replicate contaminated and uncontaminated areas can location‐specific effects be partitioned from impacts associated with radionuclide exposure (Jernfors et al., 2021; Kesäniemi, Jernfors, et al., 2019; Kesäniemi, Lavrinienko, et al., 2019). The additional discussion points raised by Antwis et al. (2021) that ‘Other studies of radiation effects in CEZ wildlife, including the microbiome studies of Lavrinienko et al. …, also have their most contaminated sampling sites within the Red Forest…’, and ‘Any study that uses the Red Forest as a location for radiation effect studies on wildlife needs to consider the historical impacts of radiation and other stressors (e.g. wildfires) on this area…’ are somewhat misleading as they neglect to consider the consistent patterns associated with radionuclide exposure in gut microbiota samples from replicate contaminated and uncontaminated locations (Lavrinienko, Mappes, et al., 2018; Lavrinienko et al., 2020). It is also relevant that Antwis et al.'s (2021) samples from *Apodemus* mice were derived from animals that had absorbed dose rates of ≥20 μGy/hr: in fact, the data from 2017 consist almost exclusively of one category of absorbed dose rate (‘high’), and yet the authors included it in analyses as categorical predictor of three levels (*n* = 0, 14 and 100 *Apodemus* in the ‘low’, ‘medium’ and ‘high’ dose rate categories respectively). The analysis by Antwis et al. (2021) potentially examines the response of *Apodemus* mice to medium and high absorbed dose rates but does not examine how exposure to radionuclides per se impacts the gut microbiota in different species of rodent. By contrast, comparable impacts of exposure to radionuclides in the gut microbiota of three (out of four) species of *Apodemus* are apparent in samples collected from the CEZ and the Fukushima accident sites (Lavrinienko, Hämäläinen, et al., 2021).

## 5 CONCLUSIONS

The literature about biological impacts of inhabiting the area surrounding the former nuclear power plant at Chernobyl is littered with arguments and counterarguments (Beresford et al., 2016; Beresford, Horemans, et al., 2020; Beresford, Scott, et al., 2020; Mousseau, 2021). Some of this discussion might be derived from simple misunderstandings in communication. For instance, Antwis et al. (2021) write ‘we present the first study of gastrointestinal (GI) tract microbiome composition in CEZ small mammals for which individual total absorbed dose rates have been estimated’. However, this statement either refers specifically to the analysis of samples from the caecum or they overlooked that previous studies of wildlife gut microbiota (faecal samples) estimated absorbed dose rates (Lavrinienko, Tukalenko, et al., 2018; Lavrinienko et al., 2020). Outside this use of language, however, several features of Antwis et al.'s (2021) study design and interpretation of data warrant more discussion.

Demonstrating that the inclusion of non‐resident fungal SV data impacts amplicon sequencing‐based analyses of ‘gut mycobiota’ has important implications for studies of wildlife gut mycobiota (Lavrinienko, Scholier, et al., 2021). A consequence of not identifying the probable resident fraction of the gut mycobiota is to promote misunderstanding about the drivers of variation in wildlife gut mycobiota (e.g. proposing macrofungi as biomarkers of a gut microbial response to radiation exposure). The inclusion of all fungal SVs can alter the pattern of interspecific differences in ‘gut mycobiota’ due to variation in ingested fungal material among host species. As the ecologies and traits of many fungi are poorly known, it can be challenging to define the authentic gut mycobiota, especially for animals with a catholic diet like the bank vole. With information about the species of fungi eaten and/or present in dietary items, it could be possible to use informatic tools (e.g. SourceTracker, Knights et al., 2011) to identify the ingested fungi. Without such data, however, the analyses of wildlife gut mycobiota will depend on the filtering decisions; for example, one may decide to focus on the macrofungal fraction (rather than plant‐associated fungi, such as endophytes, ectomycorrhizae and/or pathogens) and/or omit SVs with poor taxonomic resolution (e.g. those not assigned beyond Phylum level) (see e.g. Lavrinienko, Scholier, et al., 2021). While such filtering decisions can change with new insights into fungal biology, an educated ‘best guess’ about fungal traits presents an opportunity to stimulate new research (e.g. complementing analyses of dietary niche or identifying novel life histories of fungi). Even with imperfect information about fungal traits, informed filtering provides a more detailed assessment of the biological signal in the data than simply overlooking the ecology of fungi and animal hosts.

Understanding whether laboratory protocols and sample type impact study conclusions is an important topic in microbiota research (Ingala et al., 2018; Knight et al., 2018; Videvall et al., 2018; Zhou et al., 2020). An apparent problem with interpreting some field studies on organisms inhabiting the CEZ is derived from authors overemphasising statistically significant relationships that have little explanatory power (Beresford, Scott, et al., 2020). Given that many sources of variation in gut microbiota composition were confounded with the analysis of ‘sample type’, including a marked difference in the range of absorbed dose rates received by animals sampled for either faeces or caecum, it is hard to understand why Antwis et al.'s (2021) concluded ‘considerable variation between results for faecal and gut samples of bank voles, suggesting faecal samples are not an accurate indicator of gut composition’ or ‘… of the effect of stressors on the gut microbiome’. This interpretation of these data creates an unwarranted controversy about the effect of sample type on microbiota analyses generally, and radiation impacts on wildlife gut microbiota specifically.

With reference to studies on wildlife inhabiting the CEZ, Beresford, Scott, et al. (2020) stressed the importance of defining ‘… the scientific question as clearly and as unambiguously as possible’. Reflection on Antwis et al.'s (2021) faecal samples reinforces this need for clarity, for example about how the distribution of data affects the conclusions. With faecal samples collected from a single area that did not include animals in the low absorbed dose rate category (control animals), the Abstract, Introduction and Discussion by Antwis et al. (2021) would benefit from clear statements and hypotheses that explain (1) their sampling strategy to omit control animals (animals that did not experience elevated absorbed dose rates) and (2) that they examined the effects of absorbed dose rates of 20 μGy/hr and above on *Apodemus* mice but not the impacts of radiation exposure on gut microbiota. By contrast, with a sample of animals from contaminated and uncontaminated areas, Antwis et al.'s (2021) caecum samples from bank voles identified an association between absorbed dose rate and some taxa within the gut microbiota.

It is relevant also that the studies by Lavrinienko, Mappes, et al. (2018), Lavrinienko, Tukalenko, et al. (2018), Lavrinienko et al. (2020) and Antwis et al. (2021) employed different sampling protocols (Antwis et al. sampled animals after 1 week of pre‐baiting traps with oats and carrot/cucumber, whereas Lavrinienko et al. did not pre‐bait traps), stored their samples differently (use of ethanol or not), processed samples using different DNA extraction kits (PowerFecal DNA Isolation kit and PureLink™ Microbiome DNA Purification Kit) and collected samples in different years (2016, and 2017, 2018), from different months (Antwis et al. took samples during late July and in August, whereas Lavrinienko, Mappes, et al. (2018), Lavrinienko, Tukalenko, et al. (2018), Lavrinienko et al. (2020) collected their samples during May–July) and from different locations (Figure 1). It is essential to carefully consider the diversity of mechanisms that can elicit a change in gut microbiota (notably, season associates with a change in gut microbiota of bank voles inhabiting uncontaminated areas within the CEZ; Lavrinineko et al., 2020) when attempting to replicate a microbiota study. Without an equivalent study design, or clear explanation of why changes to the protocol were made, important drivers(s) of apparent differences among studies could be overlooked.

A comprehensive discussion of the statements presented by Antwis et al. (2021) in their abstract is important because the outcomes of studies of the wildlife inhabiting the CEZ can be used to assess the risks of radiation exposure and formulate policy. Independent examinations of the biological impacts of radionuclide exposure are needed to form robust conclusions, but these studies are informative only when the comparison incorporates an appropriate study design: failure to do so will only cloud our understanding of the biological impacts of exposure to environmental radionuclides. For the reasons outlined above, Antwis et al. (2021) made some strong assertions that are hard to reconcile with their, or indeed others', data and study design(s). One unfortunate result of this attempt to stimulate debate is yet another controversy that does not appear to be justified.

## CONFLICT OF INTEREST

The authors declare no conflict of interest.

## AUTHORS' CONTRIBUTIONS

All authors conceived the ideas; P.C.W. analysed the data; A.L. and P.C.W. led the writing of the manuscript, with all authors making critical contributions to the drafts and giving their final approval for publication.

## Data Availability

Sequence data originally presented in the study by Antwis et al. (2021) (https://doi.org/10.1111/1365‐2656.13507) are available from the NCBI SRA database under the accession number PRJNA594002.

## References

[jane13667-bib-0001] Abt, K. F. , & Bock, W. F. (1998). Seasonal variations of diet composition in farmland field mice Apodemus spp. and bank voles *Clethrionomys glareolus* . Acta Theriologica, 43, 379–389. 10.4098/AT.arch.98-49

[jane13667-bib-0002] Albenberg, L. , Esipova, T. V. , Judge, C. P. , Bittinger, K. , Chen, J. , Laughlin, A. , Grunberg, S. , Baldassano, R. N. , Lewis, J. D. , Li, H. , Thom, S. R. , Bushman, F. D. , Vinogradov, S. A. , & Wu, G. D. (2014). Correlation between intraluminal oxygen gradient and radial partitioning of intestinal microbiota in humans and mice. Gastroenterology, 147, 1055–1063. 10.1053/j.gastro.2014.07.020 25046162 PMC4252572

[jane13667-bib-0003] Antwis, R. E. , Beresford, N. A. , Jackson, J. A. , Fawkes, R. , Barnett, C. L. , Potter, E. , Walker, L. , Gaschak, S. , & Wood, M. D. (2021). Impacts of radiation on the bacterial and fungal microbiome of small mammals in the Chernobyl Exclusion Zone. Journal of Animal Ecology, 9, 2172–2187. 10.1111/1365-2656.13507 33901301

[jane13667-bib-0004] Antwis, R. E. , Edwards, K. L. , Unwin, B. , Walker, S. L. , & Shultz, S. (2019). Rare gut microbiota associated with breeding success, hormone metabolites and ovarian cycle phase in the critically endangered eastern black rhino. Microbiome, 2, 27. 10.1186/s40168-019-0639-0 PMC6377766 30770764

[jane13667-bib-0005] Barelli, C. , Albanese, D. , Stumpf, R. M. , Asangba, A. , Donati, C. , Rovero, F. , & Hauffe, H. C. (2020). The gut microbiota communities of wild arboreal and ground‐feeding tropical primates are affected differently by habitat disturbance. MSystems, 5, 1–18. 10.1128/msystems.00061-20 PMC7253362 32457237

[jane13667-bib-0006] Beresford, N. A. , Fesenko, S. , Konoplev, A. , Skuterud, L. , Smith, J. T. , & Voigt, G. (2016). Thirty years after the Chernobyl accident: What lessons have we learnt? Journal of Environmental Radioactivity, 157, 77–89. 10.1016/j.jenvrad.2016.02.003 27018344

[jane13667-bib-0007] Beresford, N. A. , Horemans, N. , Copplestone, D. , Raines, K. E. , Orizaola, G. , Wood, M. D. , Laanen, P. , Whitehead, H. C. , Burrows, J. E. , Tinsley, M. C. , Smith, J. T. , Bonzom, J. M. , Gagnaire, B. , Adam‐Guillermin, C. , Gashchak, S. , Jha, A. N. , de Menezes, A. , Willey, N. , & Spurgeon, D. (2020). Towards solving a scientific controversy – The effects of ionising radiation on the environment. Journal of Environmental Radioactivity, 211, 106033. 10.1016/j.jenvrad.2019.106033 31451195

[jane13667-bib-0008] Beresford, N. A. , Scott, E. M. , & Copplestone, D. (2020). Field effects studies in the Chernobyl exclusion zone: Lessons to be learnt. Journal of Environmental Radioactivity, 211, 105893. 10.1016/j.jenvrad.2019.01.005 30718022

[jane13667-bib-0009] Bokulich, N. A. , Kaehler, B. D. , Rideout, J. R. , Dillon, M. , Bolyen, E. , Knight, R. , Huttley, G. A. , & Gregory Caporaso, J. (2018). Optimizing taxonomic classification of marker‐gene amplicon sequences with QIIME 2's q2‐feature‐classifier plugin. Microbiome, 6, 1–17. 10.1186/s40168-018-0470-z 29773078 PMC5956843

[jane13667-bib-0010] Bolyen, E. , Rideout, J. R. , Dillon, M. R. , Bokulich, N. A. , Abnet, C. C. , Al‐Ghalith, G. A. , Alexander, H. , Alm, E. J. , Arumugam, M. , Asnicar, F. , Bai, Y. , Bisanz, J. E. , Bittinger, K. , Brejnrod, A. , Brislawn, C. J. , Brown, C. T. , Callahan, B. J. , Caraballo‐Rodríguez, A. M. , Chase, J. , … Caporaso, J. G. (2019). Reproducible, interactive, scalable and extensible microbiome data science using QIIME 2. Nature Biotechnology, 37, 852–857. 10.1038/s41587-019-0209-9 PMC7015180 31341288

[jane13667-bib-0011] Bonder, M. J. , Kurilshikov, A. , Tigchelaar, E. F. , Mujagic, Z. , Imhann, F. , Vila, A. V. , Deelen, P. , Vatanen, T. , Schirmer, M. , Smeekens, S. P. , Zhernakova, D. V. , Jankipersadsing, S. A. , Jaeger, M. , Oosting, M. , Cenit, M. C. , Masclee, A. A. M. , Swertz, M. A. , Li, Y. , Kumar, V. , … Zhernakova, A. (2016). The effect of host genetics on the gut microbiome. Nature Genetics, 48, 1407–1412. 10.1038/ng.3663 27694959

[jane13667-bib-0012] Brila, I. , Lavrinienko, A. , Tukalenko, E. , Ecke, F. , Rodushkin, I. , Kallio, E. R. , Mappes, T. , & Watts, P. C. (2021). Low‐level environmental metal pollution is associated with altered gut microbiota of a wild rodent, the bank vole (*Myodes glareolus*). Science of the Total Environment, 790, 148224. 10.1016/j.scitotenv.2021.148224 34380250

[jane13667-bib-0013] Callahan, B. J. , McMurdie, P. J. , Rosen, M. J. , Han, A. W. , Johnson, A. J. A. , & Holmes, S. P. (2016). DADA2: High‐resolution sample inference from Illumina amplicon data. Nature Methods, 13, 581–583. 10.1038/nmeth.3869 27214047 PMC4927377

[jane13667-bib-0015] Fogel, R. , & Trappe, J. (1978). Fungus consumption (mycophagy) by small animals. Northwest Science, 52, 1–31.

[jane13667-bib-0016] Guo, N. , Wu, Q. , Shi, F. , Niu, J. , Zhang, T. , Degen, A. A. , Fang, Q. , Ding, L. , Shang, Z. , Zhang, Z. , & Long, R. (2021). Seasonal dynamics of diet–gut microbiota interaction in adaptation of yaks to life at high altitude. NPJ Biofilms and Microbiomes, 7, 38. 10.1038/s41522-021-00207-6 33879801 PMC8058075

[jane13667-bib-0017] Huseyin, C. E. , Toole, P. W. O. , Cotter, P. D. , & Scanlan, P. D. (2017). Forgotten fungi — The gut mycobiome in human health and disease. FEMS Microbiology Reviews, 41, 479–511. 10.1093/femsre/fuw047 28430946

[jane13667-bib-0018] Iliev, I. D. , Funari, V. A. , Taylor, K. D. , Nguyen, Q. , Reyes, C. N. , Strom, S. P. , Brown, J. , Becker, C. A. , Fleshner, P. R. , Dubinsky, M. , Rotter, J. I. , Wang, H. L. , McGovern, D. P. B. , Brown, G. D. , & Underhill, D. M. (2012). Interactions between commensal fungi and the C‐type lectin receptor dectin‐1 influence colitis. Science, 336, 1314–1317. 10.1126/science.1221789 22674328 PMC3432565

[jane13667-bib-0019] Ingala, M. R. , Simmons, N. B. , Wultsch, C. , Krampis, K. , Speer, K. A. , & Perkins, S. L. (2018). Comparing microbiome sampling methods in a wild mammal: Fecal and intestinal samples record different signals of host ecology, evolution. Frontiers in Microbiology, 9, 1–13. 10.3389/fmicb.2018.00803 29765359 PMC5938605

[jane13667-bib-0020] Jernfors, T. , Danforth, J. , Kesäniemi, J. , Lavrinienko, A. , Tukalenko, E. , Fajkus, J. , Dvořáčková, M. , Mappes, T. , & Watts, P. C. (2021). Expansion of rDNA and pericentromere satellite repeats in the genomes of bank voles *Myodes glareolus* exposed to environmental radionuclides. Ecology and Evolution, 11, 8754–8767. 10.1002/ece3.7684 34257925 PMC8258220

[jane13667-bib-0021] Johnson, A. J. , Vangay, P. , Al‐Ghalith, G. A. , Hillmann, B. M. , Ward, T. L. , Shields‐Cutler, R. R. , Kim, A. D. , Shmagel, A. K. , Syed, A. N. , Walter, J. , Menon, R. , Koecher, K. , & Knights, D. (2019). Daily sampling reveals personalized diet‐microbiome associations in humans. Cell Host and Microbe, 25, 789–802.e5. 10.1016/j.chom.2019.05.005 31194939

[jane13667-bib-0022] Kesäniemi, J. , Boratyński, Z. , Danforth, J. , Itam, P. , Jernfors, T. , Lavrinienko, A. , Mappes, T. , Møller, A. P. , Mousseau, T. A. , & Watts, P. C. (2018). Analysis of heteroplasmy in bank voles inhabiting the Chernobyl exclusion zone: A commentary on Baker et al. (2017) “elevated mitochondrial genome variation after 50 generations of radiation exposure in a wild rodent.”. Evolutionary Applications, 11, 820–826. 10.1111/eva.12578 29875822 PMC5978973

[jane13667-bib-0023] Kesäniemi, J. , Jernfors, T. , Lavrinienko, A. , Kivisaari, K. , Kiljunen, M. , Mappes, T. , & Watts, P. C. (2019). Exposure to environmental radionuclides is associated with altered metabolic and immunity pathways in a wild rodent. Molecular Ecology, 28, 4620–4635. 10.1111/mec.15241 31498518 PMC6900138

[jane13667-bib-0024] Kesäniemi, J. , Lavrinienko, A. , Tukalenko, E. , Boratyński, Z. , Kivisaari, K. , Mappes, T. , Milinevsky, G. , Møller, A. P. , Mousseau, T. A. , & Watts, P. C. (2019). Exposure to environmental radionuclides associates with tissue‐specific impacts on telomerase expression and telomere length. Scientific Reports, 9, 850. 10.1038/s41598-018-37164-8 30696885 PMC6351625

[jane13667-bib-0025] Knight, R. , Vrbanac, A. , Taylor, B. C. , Aksenov, A. , Callewaert, C. , Debelius, J. , Gonzalez, A. , Kosciolek, T. , McCall, L. I. , McDonald, D. , Melnik, A. V. , Morton, J. T. , Navas, J. , Quinn, R. A. , Sanders, J. G. , Swafford, A. D. , Thompson, L. R. , Tripathi, A. , Xu, Z. Z. , … Dorrestein, P. C. (2018). Best practices for analysing microbiomes. Nature Reviews Microbiology, 16, 410–422. 10.1038/s41579-018-0029-9 29795328

[jane13667-bib-0026] Knights, D. , Kuczynski, J. , Charlson, E. S. , Zaneveld, J. , Mozer, M. C. , Collman, R. G. , Bushman, F. D. , Knight, R. , & Kelley, S. T. (2011). Bayesian community‐wide culture‐independent microbial source tracking. Nature Methods, 8, 761–763. 10.1038/nmeth.1650 21765408 PMC3791591

[jane13667-bib-0027] Kong, H. H. , & Morris, A. (2017). The emerging importance and challenges of the human mycobiome. Virulence, 8, 310–312. 10.1080/21505594.2017.1279780 28102762 PMC5411231

[jane13667-bib-0028] Kreisinger, J. , Bastien, G. , Hauffe, H. C. , Marchesi, J. , & Perkins, S. E. (2015). Interactions between multiple helminths and the gut microbiota in wild rodents. Philosophical Transactions of the Royal Society B: Biological Sciences, 370, 0295. 10.1098/rstb.2014.0295 PMC4528493 26150661

[jane13667-bib-0029] Lavrinienko, A. , Hämäläinen, A. , Hindström, R. , Tukalenko, E. , Boratyński, Z. , Kivisaari, K. , Mousseau, T. A. , Watts, P. C. , & Mappes, T. (2021). Comparable response of wild rodent gut microbiome to anthropogenic habitat contamination. Molecular Ecology, 30, 3485–3499. 10.1111/mec.15945 33955637

[jane13667-bib-0030] Lavrinienko, A. , Jernfors, T. , Koskimäki, J. J. , Pirttilä, A. M. , & Watts, P. C. (2021). Does intraspecific variation in rDNA copy number affect analysis of microbial communities? Trends in Microbiology, 29, 19–27. 10.1016/j.tim.2020.05.019 32593503

[jane13667-bib-0031] Lavrinienko, A. , Mappes, T. , Tukalenko, E. , Mousseau, T. A. , Møller, A. P. , Knight, R. , Morton, J. T. , Thompson, L. R. , & Watts, P. C. (2018). Environmental radiation alters the gut microbiome of the bank vole *Myodes glareolus* . ISME Journal, 12, 2801–2806. 10.1038/s41396-018-0214-x 29988064 PMC6193954

[jane13667-bib-0032] Lavrinienko, A. , Scholier, T. , Bates, S. T. , Miller, A. N. , & Watts, P. C. (2021). Defining gut mycobiota for wild animals: A need for caution in assigning authentic resident fungal taxa. Animal Microbiome, 3, 75. 10.1186/s42523-021-00134-z 34711273 PMC8554864

[jane13667-bib-0033] Lavrinienko, A. , Tukalenko, E. , Kesäniemi, J. , Kivisaari, K. , Masiuk, S. , Boratyński, Z. , Mousseau, T. A. , Milinevsky, G. , Mappes, T. , & Watts, P. C. (2020). Applying the Anna Karenina principle for wild animal gut microbiota: Temporal stability of the bank vole gut microbiota in a disturbed environment. Journal of Animal Ecology, 89, 2617–2630. 10.1111/1365-2656.13342 32939769

[jane13667-bib-0034] Lavrinienko, A. , Tukalenko, E. , Mappes, T. , & Watts, P. C. (2018). Skin and gut microbiomes of a wild mammal respond to different environmental cues. Microbiome, 6, 209. 10.1186/s40168-018-0595-0 30477569 PMC6258405

[jane13667-bib-0035] Li, F. , Li, C. , Chen, Y. , Liu, J. , Zhang, C. , Irving, B. , Fitzsimmons, C. , Plastow, G. , & Guan, L. L. (2019). Host genetics influence the rumen microbiota and heritable rumen microbial features associate with feed efficiency in cattle. Microbiome, 7, 1–17. 10.1186/s40168-019-0699-1 31196178 PMC6567441

[jane13667-bib-0036] Martinez‐Guryn, K. , Leone, V. , & Chang, E. B. (2019). Regional diversity of the gastrointestinal microbiome. Cell Host and Microbe, 26, 314–324. 10.1016/j.chom.2019.08.011 31513770 PMC6750279

[jane13667-bib-0037] Maurice, C. F. , Cl Knowles, S. , Ladau, J. , Pollard, K. S. , Fenton, A. , Pedersen, A. B. , & Turnbaugh, P. J. (2015). Marked seasonal variation in the wild mouse gut microbiota. ISME Journal, 9, 2423–2434. 10.1038/ismej.2015.53 26023870 PMC4611506

[jane13667-bib-0038] McMurdie, P. J. , & Holmes, S. (2013). Phyloseq: An R package for reproducible interactive analysis and graphics of microbiome census data. PLoS One, 8, e61217. 10.1371/journal.pone.0061217 23630581 PMC3632530

[jane13667-bib-0039] Møller, A. P. , & Mousseau, T. A. (2006). Biological consequences of Chernobyl: 20 years on. Trends in Ecology and Evolution, 21, 200–207. 10.1016/j.tree.2006.01.008 16701086

[jane13667-bib-0040] Morrison, D. J. , & Preston, T. (2016). Formation of short chain fatty acids by the gut microbiota and their impact on human metabolism. Gut Microbes, 7, 189–200. 10.1080/19490976.2015.1134082 26963409 PMC4939913

[jane13667-bib-0041] Mousseau, T. A. (2021). The biology of Chernobyl. Annual Review of Ecology, Evolution, and Systematics, 52, 87–109. 10.1146/annurev-ecolsys-110218-024827

[jane13667-bib-0042] Nguyen, N. H. , Song, Z. , Bates, S. T. , Branco, S. , & Tedersoo, L. (2015). FUNGuild: An open annotation tool for parsing fungal community datasets by ecological guild. Fungal Ecology, 20, 241–248. 10.1016/j.funeco.2015.06.006

[jane13667-bib-0043] Nilsson, R. H. , Anslan, S. , Bahram, M. , Wurzbacher, C. , Baldrian, P. , & Tedersoo, L. (2019). Mycobiome diversity: High‐throughput sequencing and identification of fungi. Nature Reviews Microbiology, 17, 95–109. 10.1038/s41579-018-0116-y 30442909

[jane13667-bib-0044] Nilsson, R. H. , Larsson, K. H. , Taylor, A. F. S. , Bengtsson‐Palme, J. , Jeppesen, T. S. , Schigel, D. , Kennedy, P. , Picard, K. , Glöckner, F. O. , Tedersoo, L. , Saar, I. , Kõljalg, U. , & Abarenkov, K. (2019). The UNITE database for molecular identification of fungi: Handling dark taxa and parallel taxonomic classifications. Nucleic Acids Research, 47(D1), D259–D264. 10.1093/nar/gky1022 30371820 PMC6324048

[jane13667-bib-0045] Oksanen, J. , Blanchet, F.G. , Friendly, M. , Kindt, R. , Legendre, P. , McGlinn, D. , Minchin, P.R. , O'Hara, R.B. , Simpson, G.L. , Solymos, P. , Stevens, M.H.H. , Szoecs, E. , & Wagner, H. (2020). vegan: Community Ecology Package.

[jane13667-bib-0046] Panek, M. , Čipčić Paljetak, H. , Barešić, A. , Perić, M. , Matijašić, M. , Lojkić, I. , Bender, D. V. , Krznarić, Ž. , & Verbanac, D. (2018). Methodology challenges in studying human gut microbiota‐effects of collection, storage, DNA extraction and next generation sequencing technologies. Scientific Reports, 8, 1–13. 10.1038/s41598-018-23296-4 29572539 PMC5865204

[jane13667-bib-0047] Park, R. , Dzialo, M. C. , Spaepen, S. , Nsabimana, D. , Gielens, K. , Devriese, H. , Crauwels, S. , Tito, R. Y. , Raes, J. , Lievens, B. , & Verstrepen, K. J. (2019). Microbial communities of the house fly *Musca domestica* vary with geographical location and habitat. Microbiome, 7, 1–12. 10.1186/s40168-019-0748-9 31699144 PMC6839111

[jane13667-bib-0048] Pickard, J. M. , Zeng, M. Y. , Caruso, R. , & Núñez, G. (2017). Gut microbiota: Role in pathogen colonization, immune responses, and inflammatory disease. Immunological Reviews, 279, 70–89. 10.1111/imr.12567 28856738 PMC5657496

[jane13667-bib-0049] Qin, J. , Li, R. , Raes, J. , Arumugam, M. , Burgdorf, K. S. , Manichanh, C. , Nielsen, T. , Pons, N. , Levenez, F. , Yamada, T. , Mende, D. R. , Li, J. , Xu, J. , Li, S. , Li, D. , Cao, J. , Wang, B. , Liang, H. , Zheng, H. , … Zoetendal, E. (2010). A human gut microbial gene catalogue established by metagenomic sequencing. Nature, 464, 59–65. 10.1038/nature08821 20203603 PMC3779803

[jane13667-bib-0050] R Core Team . (2020). R: A language and environment for statistical computing. R Foundation for Statistical Computing. https://www.R‐project.org/

[jane13667-bib-0051] Rosshart, S. P. , Herz, J. , Vassallo, B. G. , Hunter, A. , Wall, M. K. , Badger, J. H. , McCulloch, J. A. , Anastasakis, D. G. , Sarshad, A. A. , Leonardi, I. , Collins, N. , Blatter, J. A. , Han, S. J. , Tamoutounour, S. , Potapova, S. , Foster St Claire, M. B. , Yuan, W. , Sen, S. K. , Dreier, M. S. , … Rehermann, B. (2019). Laboratory mice born to wild mice have natural microbiota and model human immune responses. Science, 365, eaaw4361. 10.1126/science.aaw4361 31371577 PMC7377314

[jane13667-bib-0052] Round, J. L. , & Mazmanian, S. K. (2009). The gut microbiota shapes intestinal immune responses during health and disease. Nature Reviews Immunology, 9, 313–323. 10.1038/nri2515 PMC4095778 19343057

[jane13667-bib-0053] Sabey, K. A. , Song, S. J. , Jolles, A. , Knight, R. , & Ezenwa, V. O. (2021). Coinfection and infection duration shape how pathogens affect the African buffalo gut microbiota. ISME Journal, 15, 1359–1371. 10.1038/s41396-020-00855-0 33328653 PMC8115229

[jane13667-bib-0054] Sonnenburg, J. L. , & Bäckhed, F. (2016). Diet‐microbiota interactions as moderators of human metabolism. Nature, 535, 56–64. 10.1038/nature18846 27383980 PMC5991619

[jane13667-bib-0055] Velasco‐Galilea, M. , Piles, M. , Viñas, M. , Rafel, O. , González‐Rodríguez, O. , Guivernau, M. , & Sánchez, J. P. (2018). Rabbit microbiota changes throughout the intestinal tract. Frontiers in Microbiology, 9, 02144. 10.3389/fmicb.2018.02144 PMC6146034 30271392

[jane13667-bib-0056] Videvall, E. , Strandh, M. , Engelbrecht, A. , Cloete, S. , & Cornwallis, C. K. (2018). Measuring the gut microbiome in birds: Comparison of faecal and cloacal sampling. Molecular Ecology Resources, 18, 424–434. 10.1111/1755-0998.12744 29205893

[jane13667-bib-0057] White, T. A. , Lundy, M. G. , Montgomery, W. I. , Montgomery, S. , Perkins, S. E. , Lawton, C. , Meehan, J. M. , Hayden, T. J. , Heckel, G. , Reid, N. , & Searle, J. B. (2012). Range expansion in an invasive small mammal: Influence of life‐history and habitat quality. Biological Invasions, 14, 2203–2215. 10.1007/s10530-012-0225-x

[jane13667-bib-0058] Zaneveld, J. R. , McMinds, R. , & Thurber, R. V. (2017). Stress and stability: Applying the Anna Karenina principle to animal microbiomes. Nature Microbiology, 2, 17121. 10.1038/nmicrobiol.2017.121 28836573

[jane13667-bib-0059] Zhou, J. , Nelson, T. M. , Rodriguez Lopez, C. , Sarma, R. R. , Zhou, S. J. , & Rollins, L. A. (2020). A comparison of nonlethal sampling methods for amphibian gut microbiome analyses. Molecular Ecology Resources, 20, 844–855. 10.1111/1755-0998.13139 31990452

